# Evolution of Laboratory Diagnosis of Tuberculosis

**DOI:** 10.3390/clinpract14020030

**Published:** 2024-02-23

**Authors:** Natalia Zaporojan, Rodica Anamaria Negrean, Ramona Hodișan, Claudiu Zaporojan, Andrei Csep, Dana Carmen Zaha

**Affiliations:** 1 Doctoral School of Biomedical Sciences, University of Oradea, Str. Universitatii 1, 410087 Oradea, Romania; panus.natalia@student.uoradea.ro (N.Z.);; 2 Department of Preclinical Disciplines, Faculty of Medicine and Pharmacy, University of Oradea, P-ta 1 December 10, 410087 Oradea, Romania; 3 Emergency County Hospital Bihor, Str. Republicii 37, 410167 Oradea, Romania; 4 Department of Psycho-Neurosciences and Recovery, Faculty of Medicine and Pharmacy, University of Oradea, P-ta 1 December 10, 410087 Oradea, Romania

**Keywords:** tuberculosis, diagnosis, laboratory

## Abstract

Tuberculosis (TB) is an infectious disease of global public health importance caused by the *Mycobacterium tuberculosis* complex. Despite advances in diagnosis and treatment, this disease has worsened with the emergence of multidrug-resistant strains of tuberculosis. We aim to present and review the history, progress, and future directions in the diagnosis of tuberculosis by evaluating the current methods of laboratory diagnosis of tuberculosis, with a special emphasis on microscopic examination and cultivation on solid and liquid media, as well as an approach to molecular assays. The microscopic method, although widely used, has its limitations, and the use and evaluation of other techniques are essential for a complete and accurate diagnosis. Bacterial cultures, both in solid and liquid media, are essential methods in the diagnosis of TB. Culture on a solid medium provides specificity and accuracy, while culture on a liquid medium brings rapidity and increased sensitivity. Molecular tests such as LPA and Xpert MTB/RIF have been found to offer significant benefits in the rapid and accurate diagnosis of TB, including drug-resistant forms. These tests allow the identification of resistance mutations and provide essential information for choosing the right treatment. We conclude that combined diagnostic methods, using several techniques and approaches, provide the best result in the laboratory diagnosis of TB. Improving the quality and accessibility of tests, as well as the implementation of advanced technologies, is essential to help improve the sensitivity, efficiency, and accuracy of TB diagnosis.

## 1. Introduction

Tuberculosis (TB) is an infectious disease of global public health importance caused by the *M. tuberculosis* complex. Alarmingly, the disease has worsened with the emergence of multidrug-resistant strains of tuberculosis (MDR-TB) [1]. Annually, it is estimated that approximately 450,000 new cases of MDR-TB occur according to a report from 2021 [2].

Early diagnosis and appropriate treatment of tuberculosis are essential public health priorities. In this context, microbiology laboratories play a vital role in the rapid and accurate detection of tuberculosis and drug resistance. The latest WHO report on tuberculosis reveals the dire consequences of the COVID-19 pandemic on access to diagnosis and treatment for the disease, jeopardizing global efforts to fight tuberculosis. The significant progress made before the pandemic has been interrupted or even reversed, accentuating the challenges in achieving the set goals.

One of the significant effects of the pandemic was the decline in the global number of people diagnosed with tuberculosis. In 2020, there was an 18% reduction compared to the previous year, followed by a partial recovery in 2021. Countries such as India, Indonesia, and the Philippines recorded the steepest declines, meaning an increase in untreated cases, with a potentially fatal impact and the possibility of community spread of infection [2].

The estimated number of deaths caused by tuberculosis experienced a significant increase in the period 2019–2021, thus reversing the downward trend of the preceding years. In 2021, approximately 1.6 million deaths were recorded, of which 1.4 million were HIV-negative and 187,000 were HIV-positive. This worrying trend underscores the devastating impact of tuberculosis in the context of HIV infection [3].

There is an alarming increase in the global incidence rate of tuberculosis in the year 2021, reversing the downward trend maintained in previous decades. In parallel, there is a rise in cases of drug-resistant tuberculosis, which adds an additional layer of complexity to the management of the disease. Treatment of drug-resistant tuberculosis has also been affected by the pandemic. In 2020, the treatments experienced a temporary decrease, followed by a partial recovery in 2021. This evolution underlines the fragility of the medical system in the face of major external factors such as the COVID-19 pandemic [4,5].

To effectively treat tuberculosis, it is necessary to first make the correct diagnosis of the affected people. However, last year only 6.4 million out of a total of 10.6 million people with active TB were diagnosed and notified, which is a gap of about 40%. This gap indicates an urgent need to improve the process of TB diagnosis and access to appropriate health care [6,7].

The role of the laboratory in the diagnosis and treatment of tuberculosis (TB) is crucial. In developed countries, the use of new technologies has facilitated rapid and accurate diagnosis, identification of the causative species, and determination of drug sensitivity [8]. In recent years, molecular tests based on nucleic acid amplification techniques have been developed. They provide a rapid, sensitive, and specific diagnosis of tuberculosis and allow the determination of drug sensitivity status. These molecular techniques are currently available or being implemented in developing countries. Nevertheless, traditional diagnostic methods such as microscopy and cultures cannot yet be completely replaced. Molecular tests can be applied in parallel with these methods for the diagnosis of TB or for drug susceptibility testing. However, the application of these molecular tests is often limited by the constraints of sputum sample storage and safe transport from remote health centers to central laboratories.

Modern clinical microbiology laboratories have at their disposal a number of methods that provide an accurate and rapid laboratory diagnosis of tuberculosis. Molecular methods are now part of the diagnostic algorithm in many laboratories and have dramatically shortened the time to diagnosis [9].

Improving the accessibility and use of current diagnostic methods, including direct microscopy, culture, and drug susceptibility testing, as well as the adoption of molecular TB diagnostic technologies, should be a priority in disease control efforts [10].

Advances in molecular biology have led to the development of methods for the quick detection of *M. tuberculosis* and its drug resistance, thus providing important tools for the development of more efficient and sensitive diagnostic methods to contribute to tuberculosis control.

In countries where TB laboratory services are integrated into general laboratory services or operate as a major private sector, the question arises whether improving the quality and accessibility of laboratory services can effectively contribute to TB control or will only expand their capacity.

Recent evidence shows that the previous approach of providing separate and parallel TB laboratories was not effective enough to improve the health system. Currently, the quality of TB laboratories is increasing, and this can act as a catalyst or, conversely, as a limiting factor for other aspects of TB control [10].

Accurate and prompt diagnosis of TB is essential for the control and management of the disease, but there are numerous factors that affect the effectiveness of this process. These problems include the difficulty of correctly diagnosing latent and active forms of TB, the lack of precise protocols for different types of TB, and the difficulty of accessing diagnostic facilities in regions with limited resources [7,11].

It is essential to perform accurate drug susceptibility testing and to understand the genetic basis of *M. tuberculosis* drug resistance. Modern clinical microbiology laboratories have methods that provide accurate and rapid laboratory diagnosis of TB. However, the complications associated with MDR-TB and the increasing incidence of extremely drug-resistant (XDR) TB highlight the need for improved identification techniques for *M. tuberculosis* and drug susceptibility testing. In this regard, molecular biology has played an important role in the development of methods for the rapid detection of *M. tuberculosis* and its drug resistance. Research in the field of molecular diagnosis of tuberculosis is constantly advancing.

In addition, new treatment-resistant strains are emerging in areas where the disease was thought to have been eradicated. It is imperative to develop affordable, accurate, and rapid diagnostic technologies adapted to the needs of developing countries [12].

The aim of this paper is to review and present the history, progress, and future directions in the molecular diagnosis of tuberculosis, including the general principles, diagnostic value, advantages, and disadvantages of molecular methods used for the detection and identification of *M. tuberculosis* and associated diseases. This review provides a comprehensive study on the use of microscopic examination, solid culture medium, liquid culture medium, and molecular tests and immunological tests, highlights issues regarding TB diagnosis, and suggests future directions for the research and the development of TB diagnosis methods.

**Figure 1:**
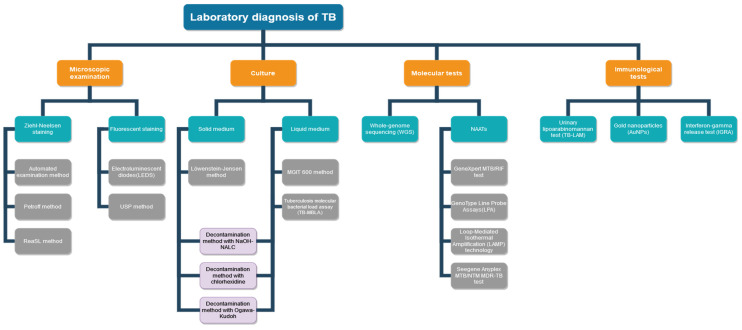
Flow diagram of the laboratory diagnosis process of tuberculosis. When, The ReaSLR method is the rapid liquefaction of sputum samples with a specific reagent called (ReaSLR), USP is universal sample processing, MGIT is mycobacteria growth indicator tube, NAAT is nucleic acid amplification test, The GeneXpert MTB RT-PCR technique is a real-time Polymerization Chain Reaction to detect the presence of *M. tuberculosis* DNA, MTB/NTM MDR-TB is method PCR multiplex Seegene Anyplex.

In Figure 1, a flow diagram is presented, highlighting the key stages of the laboratory diagnosis process of tuberculosis, including microscopic examination, culture, and molecular tests.

## 2. Microscopic Diagnosis of TB

### 2.1. Microscopic Examination with Ziehl–Neelsen Staining

Laboratory diagnosis of pulmonary tuberculosis is essential in combating the disease, with a significant role in patient management and isolation of contagious cases. In many developing countries with a high incidence of TB, advanced diagnostic methods and drug susceptibility testing are rarely available. These countries still rely on traditional methods of microscopic diagnosis and culture, but ineffective management and insufficient resources have hindered progress in (TB) control [13].

The standard method in laboratory diagnosis remains the microscopic examination of tuberculous bacilli in sputum smears, a technique that has been around for over 100 years. This provides valuable information for confirming the diagnosis and assessing the contagiousness of the patient [14,15].

Ziehl–Neelsen (ZN) staining microscopy is an essential method in the diagnosis of tuberculosis, especially where there is limited availability of facilities and equipment required for bacterial culture. This method is fast and offers the possibility to detect cases with a high bacterial load and a high risk of transmission [16,17].

**Figure 2:**
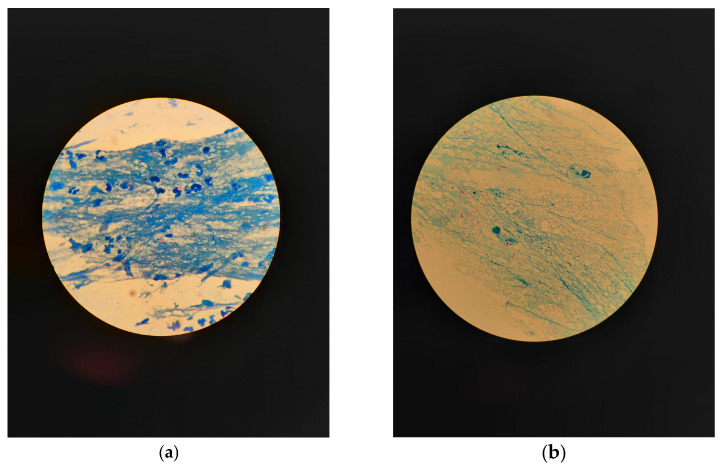
Ziehl-Neelsen staining is a specific staining technique used in microbiology laboratories to identify acid-alcohol-fast bacteria. *M. tuberculosis* in sputum. Ziehl–Neelsen staining at 1000× magnification; *M. tuberculosis* bacteria appear as thin and red bacilli. (**a**) The distinctive coloration of *M. tuberculosis* bacils is represented in red, whether they are arranged in isolation or in groups. Leukocytes and fibrin appear in a shade of blue. This smear was obtained from the patient’s sputum. (**b**) Another smear is represented, this time from a pathological product, namely bronchial lavage. Bacillus is highlighted in red, and cellular detritus is represented in blue.

The microscopic description highlights the essential details of the presence and characteristics of bacteria, relevant in the context of the diagnosis and study of *M. tuberculosis*. *M. tuberculosis* bacteria are highlighted in thin and red shades, generally with a variable length, with sizes ranging from 2 to 4 microns in length and 0.2 to 0.5 microns in width. These distinct morphological characteristics of bacteria are highlighted on a pale blue background (Figure 2).

Sputum staining is a crucial technique for the diagnosis of pulmonary tuberculosis. Although analysis of sputum stained with acid-fast fuchsin (FA) is quick and simple, it can present problems and provide incorrect results in certain clinical situations. Ziehl–Neelsen staining, on the other hand, is a more accessible technique but has its limitations, with many factors that can influence the sensitivity and specificity of the results [18].

The sensitivity of the Ziehl–Neelsen staining test in sputum smear is influenced by the number of bacteria present and it has been evaluated in different studies with a variation between 22% and 80%, requiring between 10^4^ and 10^5^ bacilli in each mL of sputum for the test to become positive [10,16,19]. However, in cases of extrapulmonary tuberculosis, co-infected with HIV, or in children, the number of bacteria may be lower than that required to obtain an optimal ZN staining [20,21].

On the other hand, a research study on a group of 544 adults with chronic cough, systematically chosen from two primary care clinics, revealed significant aspects as follows [22]. The prevalence of HIV infection in the study cohort was 83%. Tuberculosis was the most common diagnosis, being confirmed or probable in 207 HIV-positive patients (46%) and in 27 HIV-negative patients (30%). Among them, 145 HIV-positive TB patients (70%) and 20 HIV-negative TB patients (74%) had smear-positive TB cases. Only 17 HIV-positive patients and 2 HIV-negative patients were found to have smear-negative but culture-positive TB cases. Another study showed a prevalence of 37.4% of TB/HIV co-infection [23]. But it is important to note that the method is less effective in detecting tuberculosis in patients with HIV infection due to the complexity of co-infection and the need for higher diagnostic sensitivity.

In some isolated regions of low-income countries, microscopic examination of sputum smears may be the only laboratory diagnostic method available, with a diagnosis of pulmonary TB being based on clinical symptoms, chest radiography, and the results of microscopic smear tests. The procedure involves appropriate staining of mycobacteria, with the widespread use of the Ziehl–Neelsen stain, which highlights acid-fast bacilli in shades of red-purple [24,25]. 

However, this method has some limitations. It requires specialized personnel, is time-consuming, and can result in a variable sensitivity of 60% to 70% compared to the culture method. In addition, there is a risk of errors in manual reporting of results. In countries with a high prevalence of pulmonary tuberculosis, laboratory professionals may be overworked and experience fatigue from the heavy workload. Also, the staining methods used require considerable resources [26].

Some of these factors include proper sample handling, the thickness of smears, the preparation and storage of reagents, the quality of the microscopes, and the duration of staining [27]. In case of negative results, culture methods are used as a reference. In countries with a low incidence of tuberculosis, culture methods and other approaches are essential to identify and differentiate tuberculosis from other similar diseases caused by atypical mycobacteria [28]. 

In Papua New Guinea, the traditional method of sputum examination for tuberculosis involves the use of untreated sputum, a practice that presents some problems. The low sensitivity of the method and the potential risk of spreading tuberculosis bacteria in the laboratory are significant problems. In contrast, processing sputum with bleach, a more affordable and effective method, can improve the sensitivity of the test and reduce the risk of contamination in the laboratory. Thus, the use of bleach is proposed as a better, affordable, and effective alternative in sputum processing for TB diagnosis [29].

The relationship between the results of sputum smear microscopy and the time required to obtain positive results in liquid cultures was also investigated. It was found that the time required to obtain positive results is inversely proportional to the number of bacilli detected in the smears. There is also a tendency to accelerate the time to positive status, especially in the first two months of treatment. However, a significant proportion of patients continue to show acid-fast bacilli in sputum smears after two months of treatment [30].

### 2.2. Microscopic Examination with Fluorescent Staining

In high-income countries, the commonly used method includes the use of concentrated smears with fluorescent staining, which can improve the sensitivity of the test but involves higher costs [16,31]. This technique involves the use of special fluorescent dyes that bind to bacteria and emit fluorescent light under illumination of a certain wavelength [32,33]. The sensitivity of the FA method varies between 20% and 60%, and the concentrations of dyes, such as carbol fuchsin and methylene blue, are essential for the detection of *M. tuberculosis*. The WHO recommends certain dye concentrations, but there are suggestions that the use of higher concentrations may improve results in clinical settings [34,35].

Auramine staining is a more sensitive and less time-consuming technique for the diagnosis of pulmonary tuberculosis compared to the conventional ZN method. This uses the mycolic acids in the cell wall of the bacteria to retain auramine O more efficiently than the carbol fuchsin dye. Auramine O can detect *M. tuberculosis* in culture-negative specimens, which is useful in extrapulmonary tuberculosis [36].

### 2.3. The Fluorescent Microscopy Method with Electroluminescent Diodes (LEDs)

Compared to the traditional Ziehl–Neelsen staining method, LED fluorescence microscopy (FM) is more affordable, more durable, and easier to use in the medical system. It also has the advantage that LEDs have a longer lifespan and do not emit ultraviolet (UV) light, reducing the need for darkrooms and energy consumption. This method increases the sensitivity and speed of sample examination, facilitating rapid detection of *M. tuberculosis* bacteria [37,38].

A study conducted in India showed how the Revised National Tuberculosis Control Program (RNTCP) adopted LED microscopy to replace the ZN method in designated microscopy centers (DMCs) in India. A significant change in the practice of microscopy centers was observed with the adoption of fluorescence microscopy with LEDs, thus replacing the traditional ZN method [37]. However, despite the progress made, this transition does not come without challenges.

One of these notable challenges concerns the instability of fluorescent dyes, which can affect the reliability and consistency of results obtained with this new method. Moreover, an important observation is that currently there are still no standardized international guidelines to direct and ensure quality in the efficient application of LED fluorescence microscopy [37,39].

A study carried out in Tanzania in 2017 investigated the frequency of nontuberculous mycobacterial (NTM) infections among patients with a culture-negative diagnosis of pulmonary tuberculosis (TB) in countries with limited technology. From a sample of 94 patients, it was found that 26.60% had confirmed *M. tuberculosis* infections, while the rest were unconfirmed or false positive cases. In addition, cases of infections with nontuberculous mycobacteria and other bacteria were identified in culture-negative specimens. This emphasizes the importance of using advanced TB differential diagnosis techniques and rigorous clinical laboratory practices to avoid unnecessary administration of anti-TB drugs [40]. 

In Sudan, another study evaluated a new fluorescent technique compared to the ZN method for the detection of *M. tuberculosis*. The results showed that the fluorescent method is 16% more sensitive and three times faster than the ZN method [41].

Another study evaluated the effectiveness of different methods of diagnosing tuberculosis using sputum samples collected from patients suspected of having this disease. Of the 362 samples analyzed, LED-FM identified 36 samples as positive, representing 9.9%, while (ZN) identified 42 (11.6%) and GeneXpert identified 50 (13.8%). Of the samples evaluated for the presence of mycobacteria, eight were identified as nontuberculous mycobacteria (NTM). Of the remaining 332 samples, 45 (13.6%) were confirmed to have tuberculosis by culture, and 11 of these were co-infected with HIV (24.4%). The LED-FM and Gene Xpert methods showed good sensitivity and specificity in identifying tuberculosis cases [42].

### 2.4. Automated Microscopic Examination Method

To assist pathologists in TB diagnosis and to avoid the conventional time-consuming manual screening process, artificial intelligence-based methods for identifying TB in ZN staining display a variety of specificities and sensibilities. This method uses an automated microscope and specialized software to perform an operator-independent examination [43,44,45].

The automated method demonstrated a detection limit of 10^2^ bacilli/mL of sputum and a 100% positivity rate in the evaluation of sputum inoculated with *Mycobacterium bovis*, *M. bacillus* Calmette–Guerin (BCG), or *M. tuberculosis* H37Rv.

In the evaluation of 93 sputum samples, the automated method achieved a sensitivity of 97.06% and a specificity of 86.44%. This method allows the storage and reading of up to 100 smear slides and allows the export of the results into the laboratory information system. Based on the preliminary results, it is proposed to implement this automated method in the routine workflow, where only smears detected positive by examination automated microscopic examination will be confirmed by standard microscopic examination [46].

### 2.5. USP Method (Modified Auramine–Rhodamine Ziehl–Neelsen)

This method uses a fluorescence microscope to make the bacteria visible and easy to identify. The tuberculosis diagnostic method uses the dyes auramine and rhodamine, which bind to bacteria, including *M. tuberculosis*, becoming fluorescent under ultraviolet light. These bacteria are then visualized in a fluorescence microscope, highlighting them in the dark. However, the method may have limits in precision, and positive results require further confirmation by other, more precise methods, such as bacterial culture or molecular tests. A study from India published in 2014 analyzed the effectiveness of a new microscopy method called universal sample processing (USP), which has the potential to be used in laboratories with different infrastructure levels for the diagnosis of tuberculosis. Compared to the direct method and the NALC-NaOH smear microscopy method, the USP method demonstrated a marked improvement in sensitivity, identifying 18 additional positive samples, indicating the limitations of the direct smear method in detecting samples with a high bacilli load [47]. There are studies demonstrating that the use of saliva in combination with the AR fluorescent staining technique shows significant efficacy in the diagnosis and screening of patients with pulmonary tuberculosis, obtaining a positivity of 76% compared to 70% obtained by laboratory culture [19].

**Figure 3:**
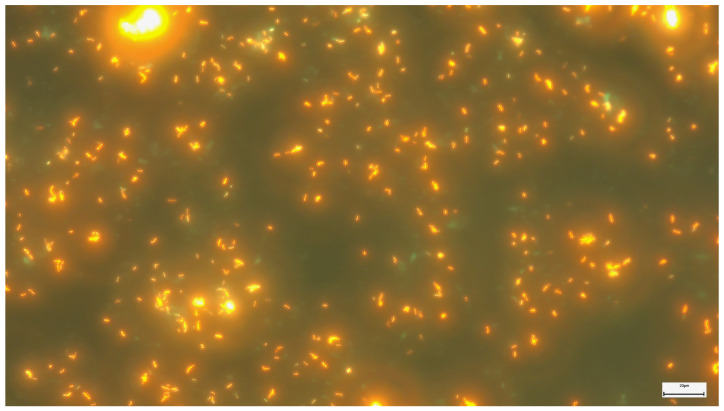
Auramine–rhodamine fluorescent stain.

Fluorescent microscopy is an efficient and fast approach to sample screening, offering remarkable advantages in terms of speed and ease of the process. This method significantly reduces the observer’s fatigue. By comparison, the modified fluorescent method demonstrated significantly higher potential in the detection of acid-resistant bacteria, especially in cases with a low concentration of bacillus. This translates into higher bacillus identification rates compared to the conventional Ziehl-Neelsen (ZN) method. In conclusion, the use of the modified fluorescent method as an adjunct to routine cytology proves to be an effective strategy for identifying acid-resistant bacteria in clinical samples [48]. Within Figure 3, which illustrates a fluorescent coloring with auramine–rhodamine, the acid-alcohol-resistant bacteria, known as acid-fast, are highlighted. The method uses a varied palette of fluorescent colors, ranging from yellow to varying shades of orange and red to highlight these particular bacteria. Acid-fast bacteria, observed in this technique, can be identified either individually, in pairs, or in small groups, and their configuration varies depending on the detected bacterial species and their specific characteristics. The background in this coloring technique is dark or black, which makes the fluorescent bacteria stand out clearly.

### 2.6. Petroff Method

This is a technique used in microbiology laboratories to concentrate and process *M. tuberculosis* bacteria from sputum samples in order to increase the efficiency and sensitivity of subsequent tuberculosis tests. The process includes the collection of sputum samples, preparation of a digestion solution to break down the samples, dilution and homogenization, centrifugation to sediment the bacteria, and decantation and resuspension of the bacteria in a concentrated solution. These concentrated bacteria are then used for various diagnostic tests, contributing to increased sensitivity and the rapid and accurate diagnosis of tuberculosis. However, compared to the modified Petroff method, the USP method did not show a significant improvement in sensitivity [49].

In addition, no notable differences in diagnostic accuracy were found between the USP method and the modified Petroff method. Regarding culture methods, both the USP and modified Petroff methods showed similar percentages of positivity, but the USP method had a higher culture contamination rate. Also, a disadvantage of the USP method is the high cost due to the use of several expensive chemicals. In conclusion, the USP method, although providing better sensitivity than the direct method and the NALC-NaOH smear microscopy method, does not provide significant improvements over the modified Petroff method. Furthermore, the USP method is associated with higher rates of crop contamination and high costs [50].

### 2.7. ReaSLR Method

This ReaSLR technique represents an innovative and affordable approach for processing sputum samples for the purpose of TB diagnosis. It is a simple and economical method that has had a significant impact on the sensitivity of smear microscopy in the detection of tuberculosis. The process involves fast liquefaction of the sputum sample with ReaSLR reagent, followed by filtration, concentration by centrifugation, and use of the resulting sediment for smear microscopy.

In a study carried out by the Department of Microbiology at the Sanjay Gandhi Institute of Medical Sciences in Lucknow, India, a total of 150 sputum samples were collected from patients with clinical suspicion of pulmonary TB; participants were in either an inpatient clinic or at an outpatient clinic within the hospital between October 2012 and January 2013. In this context, the ReaSLR method, a simple and economical technique for diagnosing tuberculosis by processing sputum, was evaluated. The results showed that compared to the modified Petroff method, the ReaSLR method achieved a higher percentage of positive results (31.33% versus 12%). With a sensitivity of 90.47% and a specificity of 91.6%, the ReaSLR method outperformed the modified Petroff method, which recorded a sensitivity of 40.47% and a specificity of 99.07%. These findings indicate that the ReaSLR method shows potential as a more sensitive and promising option for the diagnosis of pulmonary tuberculosis and could be included in tuberculosis control programs. However, it is important to conduct further studies to evaluate the effectiveness of this method on a large scale [51].

## 3. Mycobacterial Culture in TB Diagnosis

### 3.1. Lowenstein–Jensen Method (LJ)

Culture is the gold standard for the diagnosis of tuberculosis and a highly specific and sensitive detection method that can increase positivity by up to 30% in cases of negative sputum smear microscopy, providing definitive confirmation of the presence of *M. tuberculosis* and further allowing to perform drug susceptibility testing [52]. The Löwenstein–Jensen (LJ) culture technique involves taking a biological sample, such as sputum, which is then spread evenly over the surface of the solid medium. The medium is then kept under controlled temperature and humidity conditions, allowing the mycobacteria to grow and form characteristic colonies.

These colonies can then be examined under a microscope to identify the presence of specific tuberculosis bacteria. Colony development and morphology can provide essential information for accurate diagnosis. Despite its increased sensitivity compared to the examination of smears for acid-fast bacilli (80–85%), the LJ method is time-consuming, with results available after an incubation of 4–6 weeks [53]. False positive MTB cultures are rarely questioned, but rates vary from 2% to 4%. Strict adherence to laboratory techniques and recognizing the possibility of false positive MTB cultures, especially when they are not consistent with clinical data, are essential in preventing the misdiagnosis of tuberculosis [54,55].

A study conducted in Indonesia for one year aimed to evaluate the performance of Thin-Layer Agar T (TLA) culture in the diagnosis of tuberculosis compared to the LJ culture method. The sensitivity of TLA was significantly higher than for LJ with a median time to detection significantly shorter, so TLA has been proposed as an equivalently sensitive but faster alternative compared to the traditional method on LJ [56].

**Figure 4:**
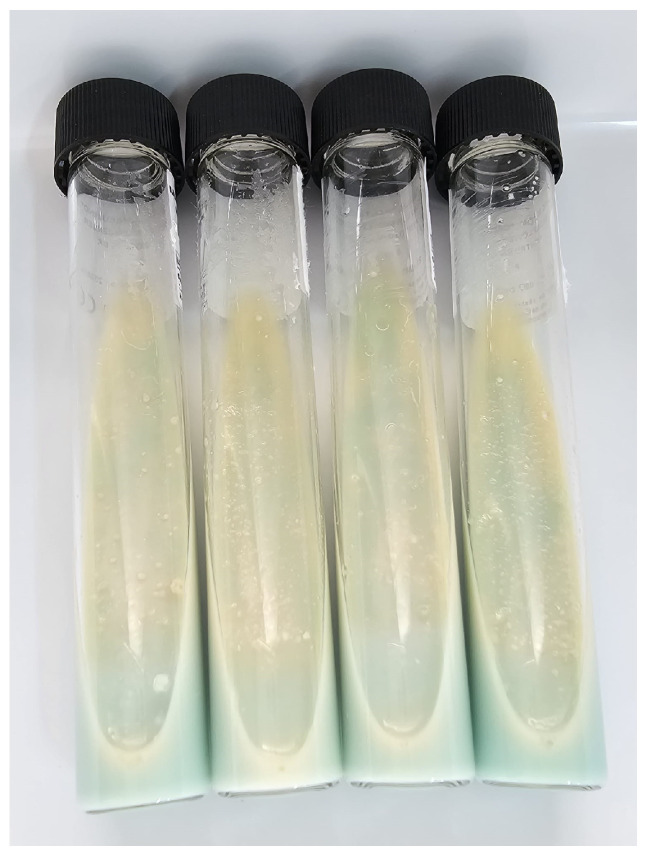
Lowenstein–Jensen medium (LJ) characteristic colonies of *M. tuberculosis*.

Figure 4 provides a visual representation of the Lowenstein–Jensen (LJ) culture environment dedicated to the cultivation of *M. tuberculosis*. In this figure, distinct and characteristic colonies of the bacterium are highlighted, which begin to develop within 4–6 weeks of incubation. Colonies grown in the Lowenstein–Jensen and *M. tuberculosis* environments stand out for their rugged texture and distinctive yellow-white coloration, thus providing an effective way to visually identify the mycobacteria.

### 3.2. MGIT 960 Technology

The MGIT 960 technique (Mycobacteria Growth Indicator Tube 960) is an advanced and automated method for the detection and cultivation of the bacterium *M. tuberculosis*. This innovative technique was developed to improve the efficiency and speed of the diagnostic process, allowing the growth of mycobacteria in special liquid media in a controlled and monitored system. Biological samples are collected from patients with suspected tuberculosis and then treated to eliminate possible contaminants, ensuring a uniform suspension of bacteria. This bacterial suspension is placed in special culture tubes called “MGIT tubes”, which contain an optimal liquid medium with nutrients and a fluorescent indicator. The MGIT tubes are inserted into an automatic device, the BACTEC™ MGIT™ 320 instrument (Becton Dickinson, Franklin Lakes, NJ, USA), which continuously monitors the bacteria. As the bacteria multiply, they consume nutrients and produce gases, which leads to increased fluorescence and detection in real time [57].

The BACTEC system Mycobacteria Growth Indicator Tube (MGIT) (Becton Dickinson, Taipei, Taiwan) is a sensitive, safe, and automated liquid culture solution. However, the short time to a positive result is not reflected in the rapid identification of mycobacteria in liquid cultures. Typically, the Mycobacterium complex tuberculosis (MTBC) and nontuberculous mycobacteria (NTM) from positive MGIT cultures for growth and acid-fast (AFB) (hereafter referred to as positive MGIT cultures) are identified by subculture on solid media. MTBC in liquid cultures can be identified in approximately 15 min through a variety of immunochromatographic tape tests [58].

Based on a thorough review of the scientific evidence and advice from experts in the field, this recommendation led to the adoption of liquid culture media as the reference standard in the diagnosis of tuberculosis. Although these provide faster results (approximately 10 days), liquid media are more susceptible to contamination and require special precautions to prevent cross-contamination [59]. The National Reference Laboratory, from Colombia National Laboratory Network and the National Institute of Health and Public Health Laboratories of Antioquia, carried out a comparative study between the method BACTEC™ MGIT™ 960 and the nitrate reductase (NRA) assay versus the ratio method on the Löwenstein–Jensen medium. The aim was to evaluate resistance to antituberculosis drugs. Of the 183 Mycobacterium tuberculosis isolates analyzed, MGIT 960 revealed a sensitivity and specificity of 90% for isoniazid (INH) and 100% and 99.4% for rifampicin (RMP), respectively. NRA showed a sensitivity and specificity of 86% and 94.7%, respectively, for INH and 100% and 99% for PMR [60].

### 3.3. Decontamination Method with NaOH-NALC

Sample decontamination is a crucial step in the preparation of biological samples before they are inoculated on Lowenstein–Jensen medium and liquid MGIT medium, distinct techniques used in the diagnosis of tuberculosis. Despite the fact that solid media are more resistant to contamination, the waiting time for obtaining results is higher. Also, liquid systems and drug susceptibility testing are more complex and sensitive but require rigorous control of contamination and isolation of nontuberculous mycobacteria [53].

Decontamination with the (NaOH-NALC) method is an essential step in the TB diagnosis process, but it is important to emphasize that the use of N-acetyl-L-cysteine (NALC) and sodium hydroxide (NaOH) can negatively influence recovery and viability bacteria in the samples.

This finding emphasizes the need for a careful approach to diagnostic and treatment methods [61,62]. The conclusions obtained from the study indicate that the use of the (NALC-NaOH) method leads to a decrease in the efficiency in the recovery of *M. tuberculosis* bacteria from the samples. This finding is consistent with results obtained in previous studies, which observed a decrease in the recovery rate of approximately 20%. This emphasizes the need to adopt molecular diagnostic techniques for the precise identification of this bacterium [63].

Another article from Baltimore, Maryland, presented a new sample processing method for more efficient detection of mycobacteria. This method used a substance called C18-carboxypropylbetaine (CB-18) and had increased sensitivity in both smear tests and cultures. Additional parameters of the CB-18 method were studied, showing that eliminating an incubation step further improved the results of the culture assays. The CB-18 method was compared with two other current processing methods (NALC-NaOH and Tween 80) for the recovery of mycobacterial isolates. The CB-18 method achieved the best results, with an average recovery of tuberculous isolates of 86% and nontuberculous isolates of 73% [63].

Another study conducted reveals that long transport of samples to culture laboratories can lead to contamination and substantial loss of viability, thus impacting bacterial culture results. Cetylpyridinium chloride (CPC) transport medium using Difco buffer led to a marked improvement in culture results [64,65].

As a result, the transport of samples in CPC followed by decontamination with NALC-NaOH and neutralization with Difco buffer, gave the best culture results, both on liquid MGIT and on LJ media. These findings may lead to improved sample processing methods in tuberculosis diagnostic laboratories [65].

### 3.4. Tuberculosis Molecular Bacterial Load Assay (TB-MBLA)

It is a molecular bacterial burden assay to measure the loss of viability of *M. tuberculosis* following treatment with NALC-NaOH of *M. tuberculosis* H37Rv pure culture and clinical sputum samples from patients with pulmonary TB.

Following this study, the impact of NALC-NaOH use on MTB was investigated. By comparing this traditional method with a modern technique called MBLA, the researchers found that the use of NALC-NaOH leads to a significant decrease in the detectable bacterial load. This phenomenon was associated with a reduction in the number of colony-forming units (CFU) per milliliter and the number of viable cultures on solid media. Moreover, the use of NALC-NaOH has been observed to prolong the time required to obtain positive results in liquid cultures [66].

### 3.5. The Method of Decontamination with Chlorhexidine

Another chlorhexidine method was shown to be less harmful to the viability of *M. tuberculosis* bacteria compared to the NALC-NaOH method. This allows better recovery of intact and viable bacteria from samples, which increases diagnostic sensitivity and accuracy.

A study conducted by the Institute Hospitalo-Universitaire of Marseille University in France focused on evaluating the effectiveness of chlorhexidine in the decontamination of sputum samples for MTB cultivation. By testing different concentrations of chlorhexidine (0.1%, 0.5%, and 0.7%) on a sample of 191 clinical samples, the researchers identified that a concentration of 0.7% chlorhexidine recorded a contamination rate of 0%. These results suggest that chlorhexidine is an effective option for decontaminating sputum samples for MTB isolation, providing a reliable method of sample preparation for subsequent culture and detection [64].

### 3.6. Decontamination Method with Ogawa-Kudoh

The Ogawa-Kudoh culture medium decontamination and inoculation method allows bacteria to be sampled, decontaminated, and cultivated in a controlled environment, facilitating the identification and confirmation of the presence of tuberculosis.

This culture medium contains agar, malt extract, glycerol, and other nutrients that favor the growth of *M. tuberculosis*. It has a softer texture and is often used for the rapid cultivation and observation of bacterial colonies.

In a study conducted in Brazil, the two decontamination methods, respectively, Ogawa-Kudoh and modified Petroff, were compared on a sample of 205 sputum samples taken from 166 patients. The results indicated that both methods demonstrated efficiency in the detection of mycobacteria, with no significant differences between them in terms of results or culture contamination rate. The conclusion of this study emphasized the excellent agreement between the two decontamination techniques [65].

## 4. Molecular Methods

### 4.1. Conventional Nucleic Acid Amplification Tests (NAAT)

Several studies have demonstrated the utility of molecular biology tests for the diagnosis of tuberculosis. These nucleic acid amplification tests (NAATs) have demonstrated a sensitivity of 81%, offering higher performance, especially in cases of patients with obvious symptoms of active tuberculosis [67].

**Figure 5:**
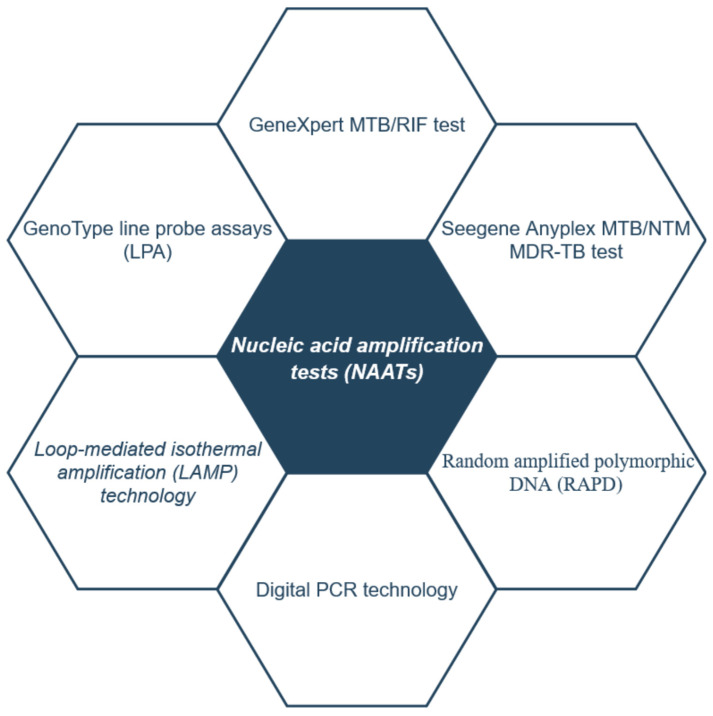
Conventional and widely used NAATs.

In Figure 5, conventional nucleic acid amplification tests are shown that are used in the molecular diagnosis of *M. tuberculosis*. These tests are essential for the rapid and accurate detection of tuberculosis and are used in diagnostic laboratories.

The GeneXpert MTB/RIF test uses real-time PCR technology for simultaneous detection of *M. tuberculosis* and rifampicin resistance, one of the key medicines in the treatment of tuberculosis.

Methods of isothermic nucleic acid amplification (NAAT) for drug resistance detection such as the GenoType MTBDRplus allow the detection of genetic mutations associated with resistance to anti-TB drugs, including rifampicin and isoniazid.

Multiplex PCR allows the simultaneous detection of several pathogens or different genotypes of *M. tuberculosis*. Multiplex PCR can be useful in cases where there is a suspicion of co-infection or when specific pins are wanted to be identified.

Isothermic nucleic acid amplification (LAMP) is a DNA-specific sequence amplification technique developed to detect and amplify genetic material effectively and quickly at a constant temperature. As an isothermic technique, LAMP is suitable for use in locations with limited resources where complex equipment or laboratory conditions are not normally available.

Random DNA polymorphism amplification (RAPD) is a technique that uses short, nonspecific DNA sequences to amplify genomic DNA. It can be used to highlight genetic variations between different strains of *M. tuberculosis*.

Digital PCR technology allows accurate detection and quantification of the amount of DNA present in a sample. Digital PCR can be useful in monitoring treatment responses and in the early detection of relapses.

Biosensors and nanoparticles are last-generation technology, and their use in the detection of tuberculosis involves innovative technologies for the specific identification of bacterial components at the molecular level.

New molecular technologies are now available for the rapid screening of TB drug resistance. The World Health Organization (WHO) has approved several molecular tests, such as the GenoType MTBDRplus line probe LPA test and GeneXpert MTB/RIF, to detect MDR-TB. In many countries, especially those with limited resources, these molecular tests are only available in reference laboratories [1].

#### 4.1.1. GenoType Line Probe Assays (LPA)

Molecular tests such as LPA are considered ideal for rapid diagnosis and can be used directly on diagnostic samples. LPA uses nuclei acid amplification techniques such as PCR and reverse hybridization to rapidly detect drug resistance mutations.

Two affordable LPA tests are INNO-LiPA Rif TB and GenoType MTBDRplus. INNO-LiPA Rif TB was introduced by Innogenetics and has been approved by the WHO, with high sensitivity and specificity for the identification of *M. tuberculosis* bacteria and drug resistance mutations [68].

GenoType MTBDR, introduced by Hain Lifescience, has a sensitivity and specificity of 99% and 100% for rifampicin resistance and 88.4% and 100% for isoniazid resistance. MTBDRplus, an improved version, has been validated by the WHO and has shown outstanding analytical efficacy for the identification of multidrug-resistant tuberculosis (MDR-TB).

The GenoType MTBDRplus Test Version 2.0, approved by the WHO in 2012, allows instant identification of mutations and has a high recognition rate of rifampicin resistance. These molecular tests have made significant improvements in the diagnosis and identification of drug resistance in tuberculosis [16,69].

A retrospective analysis investigated the effectiveness of the sample amplification tests in the detection of *M. tuberculosis* complex and the diagnosis of multidrug-resistant tuberculosis (MDR-TB). The laboratory at the Microbiology Department of the Bhopal Memorial Hospital and Research Center in Madhya Pradesh, India, examined sputum samples from patients with suspected tuberculosis. Of the 1294 acid-fast bacilli AFB-positive sputum samples tested with LPA, *M. tuberculosis* complex was detected in 94.04% of the samples but not identified in 5.9% of them [70].

Also, 5.1% of sputum samples were found to be negative for *M. tuberculosis* complex by LPA and culture. In a small percentage of AFB-positive samples, *M. tuberculosis* complex could not be identified by LPA, even if confirmed by culture. These results highlight the limitations of the LPA test in detecting *M. tuberculosis* in certain sputum samples and highlight the importance of using multiple diagnostic methods for a complete and accurate assessment of MDR-TB [70].

Another study from Nigeria analyzed a total of 67 gastric samples and 31 sputum samples to assess the presence of *M. tuberculosis* in children. The *M. tuberculosis* detection method by sandblasting microscopy (SM) was found to provide positive results in 3.0% of gastric samples and in 16.1% of sputum samples. In contrast, the use of polymerase chain amplification (LPA) detected *M. tuberculosis* in 41.8% of gastric samples and in 58.1% of sputum samples [71].

Comparing these results with other similar studies, it was observed that the LPA method had a higher yield in detecting *M. tuberculosis* in sputum samples and the SM method provided poorer results overall. In addition, it was found that the use of LPA enabled the detection of *M. tuberculosis* in sputum samples that were initially negative for the SM test. These findings suggest that the use of the LPA method may improve the diagnosis of pulmonary tuberculosis in children, providing a higher sensitivity in identifying *M. tuberculosis* in sputum samples [71].

Another study conducted in Central India looked at the 1528 sputum samples analyzed and found that 1294 were positive in the microscopic (smear) test and 234 were negative in this test. Of the 1294 LPA tests performed, 77 samples (5.9% of the total) did not show the specific band for the *M. tuberculosis* complex. Of the samples with the TUB band present (1217 samples), different types of drug resistance were identified. A total of 67 (5.1%) sputum samples were negative for *M. tuberculosis* complex by both LPA and culture [70].

The performance of the GenoType MTBDRplus assay is comparable to that of the GeneXpert MTB/RIF assay. A meta-analysis showed excellent sensitivity and specificity for the detection of resistance to isoniazid, rifampicin, and MDR-TB. However, the sensitivity of the test may vary, being higher in smear-positive cases and lower in smear-negative cases. Also, the test may have a high percentage of invalid results for direct smear-negative sputum samples [72].

#### 4.1.2. Multidrug-Resistant Tuberculosis (MDR-TB)

The importance of molecular tests in the laboratory diagnosis of multidrug-resistant tuberculosis (MDR) represents a significant challenge due to long-term treatment, limited options, and high costs, with a significant percentage of cases leading to treatment failure and death. Rapid diagnosis and an early start of treatment are essential to reduce the spread of the infection. Various research has highlighted the usefulness of molecular biology tests in the diagnosis of multidrug-resistant tuberculosis (MDR).

The MDRTDR Plus test is a method for the rapid detection of rifampicin (RIF) and isoniazid (INH) resistance, which includes rpoB probes used for rifampicin resistance detection (RIF) along with katG and inhA probes, which are used for identifying high-level and low-level isoniazid resistance (INH), respectively [73].

Even if traditional drug sensitivity testing (DST) is the standard, it can take weeks. Liquid cultivation methods are faster but involve high costs. The results obtained with GenoType MTBDRplus for resistance to rifampicin (RIF) and isoniazid (INH) are comparable to those of the conventional method, DST [74].

In one study, the efficiency of the line probe test (GenoType MTBDRplus) was compared with the results obtained by solid culture (LJ). The test successfully identified resistance to rifampicin in 70 out of 71 cases, to isoniazid in 86 out of 93 cases, and MDR-TB in 66 out of 68 cases, showing a 96% overall correlation. Sensitivity and specificity reached high levels, respectively, 98% and 99% for rifampicin, 92% and 99% for isoniazid, and 97% and 100% for MDR-TB. The frequencies of katG, inhA, and combined mutations were 83%, 11%, and 6%, respectively [75].

In another study conducted in Ethiopia, resistance to medication in the case of tuberculosis was analyzed in 274 patients using sputum samples and the MTBDRplus test. The results reveal the presence of drug-resistant tuberculosis (MDR-TB) in 72 samples, with remarkable test sensitivity and specificity.

The diagnostic performance of Genotype MTBDRplus in a direct positive sputum sample was sensitive and specific, facilitating early detection of MDR-TB. However, in the case of negative sputum samples with direct smear, the test’s effectiveness decreased, highlighting an increased level of invalid results in the detection of *M. tuberculosis* and resistance to MRI and/or INH. Thus, the implementation of the MTBDRplus VER 2.0 test for MDR-TB detection in direct negative smear samples could be limited in the effective use of this test [76].

#### 4.1.3. Extensively Drug-Resistant Tuberculosis (XDR-TB)

Extremely drug-resistant TB (XDR-TB) is an advanced and extremely dangerous form of tuberculosis. XDR-TB is defined as a form of tuberculosis that is resistant to at least four of the most effective anti-TB drugs, including the two first-line drugs (isoniazid and rifampicin), at least one drug in the aminoglycoside class (such as amikacin, kanamycin, or capreomycin), and, at most, one medicine in the fluoroquinolone class (such as levofloxacin and moxifloxacin).

Drug-resistant strains of *M. tuberculosis* are formed as a result of spontaneous chromosomal mutations, but they can also be induced by the improper use of anti-TB drugs, significantly contributing to the development of drug-resistant TB.

Primary or secondary direct transmission, as well as inadequate treatment of tuberculosis over a long period of time, can lead to the development of highly resistant TB (XDR-TB) among patients [77].

Treatment of multidrug-resistant tuberculosis (MDR-TB) requires the administration of fluoroquinolones (FQ) and injectable drugs. FQs, broad-spectrum antibacterial agents against *M. tuberculosis*, act by inhibiting mycobacterial DNA gyrase, preventing the relaxation and reproduction of bacterial ADN.

Fluoroquinolone resistance in tuberculosis is often generated by mutations in the genes that encode the gyrase subunits, especially *gyrA* and *gyrB*. Frequent mutations occur in a preserved region of the *gyrA* gene (codons 74–113) and less frequently in the *gyrB* gene (461–499), called the determining region of quinolone resistance (QRDR).

Injectable drugs, such as kanamycin (KAN), amikacin (AMK), and capreomycin (CAP), are antibiotics that inhibit protein synthesis. Cross-resistance to these second-line drugs (AMK, KAN, and CAP) is conferred by mutations in the *rrs* gene, which codes RNAr 16S [78].

The GenoType MTBDRsl is a test for the identification of resistance to fluoroquinolones (FQs; ofloxacin, moxifloxacin, and levofloxacin) and second-line injectable drugs (SLIDs; amikacin, kanamycin, and capreomycin) in the case of extensively drug-resistant forms of tuberculosis (XDR-TB) [79].

The detection of resistance to fluoroquinolones and aminoglycosides by molecular methods is becoming increasingly complex given the cross-resistance between these drugs. The molecular characterization of resistance was achieved by sequencing the *gyrA* gene DNA for fluoroquinolone (FQ) and using the polymerase chain reaction technique (PCR) and the PCR restriction fragment length polymorphism (RFLP) for *rrs* genes associated with aminoglycosides [80].

In one study, the diagnostic accuracy of the MTBDRsl test for the detection of fluoroquinolone resistance (FQ), injectable drug resistance (SLID), and extensively drug-resistant tuberculosis (XDR-TB; defined as MDR-TB plus resistance to an FQ and an SLID) was evaluated. Testing was carried out both indirectly (on cultures confirmed to be positive for TB) and directly (on sputum samples positive for smear).

Following this study, the findings show that the MTBDRsl test indicates fluoroquinolone (FQ) resistance, providing confidence for second-line treatment, including injectable drug resistance (SLID) and extensively drug-resistant tuberculosis (XDR-TB). However, the test does not detect approximately one in five cases of FQ resistance and does not identify about one in four cases of SLID resistance. With regard to kanamycin resistance, MTBDRsl has the lowest sensitivity of the three SLID drugs. The test can omit between one in four and one in three cases of TB-XDR. The diagnostic accuracy of MTBDRsl is similar when using culture isolates or positive sputum smears. Due to the variation in the location of mutations between strains, further research is needed to assess the accuracy of tests in different contexts. Although MTBDRsl can be chosen as the initial test due to its reliability and speed, a negative result may justify the conduct of additional tests, according to the decision of clinicians [79].

### 4.2. Methods Based on Real-Time Genetic Amplification Technology RT-PCR GeneXpert MTB/RIF

The Xpert MTB/RIF assay is an automated, cartridge-based, easy-to-use system with a closed amplification system to prevent cross-specimen contamination. This test can be easily performed by laboratory technicians without the need for advanced biosafety equipment. In February 2015, the FDA approved the expanded use of the Xpert MTB/RIF test to reduce the isolation period of patients with suspected tuberculosis. According to the new guidelines, one or two negative Xpert MTB/RIF test results are sufficient to rule out pulmonary tuberculosis, in contrast to previous CDC recommendations that required isolation until three consecutive negative AFB smear results from sputum to rule out contagious tuberculosis [16]. The GeneXpert system is an innovative molecular diagnostic platform used for the rapid detection of *M. tuberculosis*. This system features an interface that allows users to enter data, interpret results, and monitor test progress. The MTB/RIF cartridge contains the necessary elements for the amplification and detection of the specific DNA of the bacterium *M. tuberculosis* and for the identification of mutations associated with rifampicin resistance. Through the cartridge, the MTB/RIF test can be performed quickly and without requiring any laborious preparation, which makes it suitable for use in field laboratories or in resource-limited areas, contributing to the diagnosis and management of the disease in an efficient manner.

The Xpert MTB/RIF test is a fully automated PCR test based on PCR in real time that detects *M. tuberculosis* and mutations associated with resistance to rifampicin (RIF), the 81 bp basic region of the *rpoB* gene [81]. The analytical sensitivity of the Xpert MTB/RIF test is to five genomic copies of purified DNA and 131 CFU·mL^−1^ of *M. tuberculosis* in sputum. There was not any cross-reactivity with nontuberculous mycobacteria (NTM) detected [82].

Multinational evaluations have confirmed the feasibility, accuracy, and effectiveness of the Xpert MTB/RIF test in healthcare facilities in tuberculosis-endemic countries in Africa, Asia, and Latin America. These results have led the World Health Organization to support the expanded use of this technology. Initially, the WHO recommended the use of the Xpert MTB/RIF test for patients with suspected multidrug-resistant tuberculosis (MDR-TB) and those with HIV co-infection.

Recently, the WHO recommended that programs move away from the use of smear microscopy and prioritize the initial use of the Xpert MTB/RIF test. The sensitivity and specificity of the Xpert MTB/RIF test for the diagnosis of pulmonary tuberculosis in adults are approximately 90% and 98%, respectively, using culture as the reference standard [83].

Several studies have investigated the possibility of using the Xpert MTB/RIF test instead of direct smear microscopy as the primary screening method for urgent clinical samples in a context characterized by a low prevalence of tuberculosis [81].

GeneXpert MTB/RIF, a test subject to numerous studies and validations in various clinical settings, has been shown by a meta-analysis in low- and middle-income countries to have a high aggregated sensitivity and specificity as an initial test replacing smear microscopy. With a sensitivity of 89% and a specificity of 99%, it has proven effectiveness. However, the sensitivity of the test was higher in cases with positive results on microscopy than in those with negative results. Among people without HIV, the sensitivity was 86%, while for those with HIV, it was 79% [72].

In the context of extrapulmonary tuberculosis, a condition affecting organs and tissues outside the lungs, the performance of the GeneXpert MTB/RIF system was evaluated. The study included a considerable number of patients with various forms of extrapulmonary tuberculosis, such as nodular, peritoneal, articular bone, and genitourinary tuberculosis. The GeneXpert MTB/RIF system was compared to the standard reference method, which involves culturing the bacterium and identifying it by traditional laboratory methods [84].

Extrapulmonary tuberculosis (EPTB) accounts for approximately one-fifth of the total cases of tuberculosis in immunocompetent patients. The incidence of EPTB is significantly increased in HIV-positive people, exceeding 50% of all tuberculosis cases associated with this condition. Despite the fact that molecular methods generally do not reach the expected level in the diagnosis of tuberculosis, the polymerase chain reaction technique (PCR) is proving to be a particularly useful tool in the diagnosis of EPTB and may also be used to identify drug-resistant strains [85,86]

In a study conducted at the Bacteriology Department of the Mohammed Military Hospital, Morocco, 714 patient samples were analyzed. The mean age was 47.21 ± 19.98 years, with the majority being male (66.4%). Of the total of 714 samples, 285 came from the lungs and 429 from other areas of the body. The positive detection rate by microscopy was 12.88%, by GeneXpert MTB/RIF, it was 20.59%, and by culture, it was 15.82%. For lung samples, positive detection rates were higher: 18.9% by microscopy, 23.85% by GeneXpert MTB/RIF, and 20.35% by culture. For extrapulmonary samples, the rates were lower: 9.71% by microscopy, 18.41% by GeneXpert MTB/RIF, and 12.82% by culture. GeneXpert MTB/RIF showed a sensitivity of approximately 78.2% and a specificity of 90.4% in both sample types, while for extrapulmonary samples, these figures were 79.3% for sensitivity and 90.3% for specificity [87].

A study was performed using two diagnostic methods: ZN smear and GeneXpert MTB/RIF test. The research focused on tuberculous meningitis (TBM), a severe form of tuberculosis. The GeneXpert MTB/RIF test was evaluated for the diagnosis of TBM in a large group of patients in Vietnam. Although the Ziehl–Neelsen smear remained the most sensitive technique, the GeneXpert MTB/RIF test made an important contribution to the early diagnosis of tuberculous meningitis [88,89,90].

Another study conducted in Korea analyzed the effectiveness of the Xpert MTB/RIF Test and an MTB nested PCR in the identification of *M. tuberculosis*. Clinical lung and nonpulmonary samples were collected from 171 patients with suspected tuberculosis. The results showed that the sensitivity, specificity, positive predictive value (PPV), and negative predictive value (NPP) of the Xpert Test MTB/RIF for the diagnosis of tuberculosis with *M. tuberculosis*-positive culture were 86.1%, 97.8%, 91.2%, and 96.4%, respectively. In comparison, values of the nested PCR were 69.4%, 94.1%, 75.8%, and 92.0%, respectively. In addition, the Xpert MTB/RIF test demonstrated a significantly longer response time compared to nested PCR, with a median of 0 [0–4] days versus 4 [1–11] days, respectively (*p* under 0.001) [91].

Another study from Malaysia published in 2021 was carried out to reduce the underdiagnosis of pulmonary tuberculosis with negative ZN smear results, and the clinical and radiological characteristics of patients with this form of tuberculosis were evaluated. The research included 235 patients from a clinic in Luyaun between September 2016 and June 2017. Of the 50 cases of pulmonary tuberculosis with smear-positive results, 49 samples were confirmed positive by the Gene-Xpert MTB/RIF test and by cultivation (MTB). In contrast, of the 185 cases with pre-summative negative smear results, the Gene-Xpert MTB/RIF test identified 21 positive cases. These results were confirmed by MTB cultivation. Compared with the traditional method of detecting acid-fast bacilli in sputum, the Gene-Xpert MTB/RIF test showed higher sensitivity and specificity with almost complete accuracy. This research highlights the importance of using the Gene-Xpert MTB/RIF test in the rapid and accurate diagnosis of pulmonary tuberculosis, thereby contributing to the reduction in underdiagnosis and the initiation of early treatment for this disease [92,93].

Also, research from the comparative study analyzed the methods of rapid diagnosis of tuberculosis recurrence. Detection of recurrence can be challenging given that *M. tuberculosis*-specific DNA can be persistently present in sputum and bronchopulmonary samples, even when the disease is not active [94]. Further development of molecular tests included lowering the threshold of detection (via Xpert MTB/RIF Ultra technology) and additional gene analysis associated with resistance, resulting in significant improvement in the diagnosis of tuberculosis including in children [95,96].

In a study, the diagnostic accuracy of the detection of *M. tuberculosis*-specific DNA by either the Gene-Xpert MTB/RIF technique or the *M. tuberculosis*-specific ELISPOT method in bronchoalveolar lavage (BAL) samples was compared with the results of *M. tuberculosis* culture from sputum or bronchopulmonary samples in patients with suspected recurrence of pulmonary tuberculosis. Among the 44 patients with a history of tuberculosis and suspected recurrence of the disease, only 4 of them (9.1%) were confirmed to have recurrent tuberculosis by the culture method. As for the Gene-Xpert MTB/RIF method, it was able to detect *M. tuberculosis* DNA in the bronchoalveolar lavage in one of four patients with recurrence (25%), as well as in two of forty patients (5%) with previous tuberculosis without recurrence. In contrast, the BAL-ELISPOT technique, using a threshold of >4000 target-specific early antigenic lymphocytes 6 or culture-filtered protein-specific interferon γ 10, provided positive results for all four patients with recurrence (100%) and for two of forty patients (5%) with previous TB without recurrence [97].

In a study carried out at the Department of Bacteriology, Mohammed V Military Teaching Hospital/Faculty of Medicine and Pharmacy University Mohamed V, Rabat, a total of 714 samples were examined: 285 were taken from the lungs and 429 from other areas of the body. The diagnostic methods used, microscopic examination (ZN), GeneXpert MTB/RIF, and bacterial culture, had variable infection detection rates depending on the type of samples (pulmonary or extrapulmonary). Positive detection rates for microscopy (ZN), GeneXpert MTB/RIF, and culture were 12.88%, 20.59%, and 15.82%, respectively. In detail, for lung samples, these rates were 18.9%, 23.85%, and 20.35%, and for extrapulmonary samples, they were 9.71%, 18.41%, and 12.82%. The GeneXpert MTB/RIF assay showed close sensitivity and specificity in pulmonary (78.2% and 90.4%) and extrapulmonary (79.3% and 90.3%) samples [87].

Despite the progress made, challenges are still encountered in efforts to develop more accurate methods for the diagnosis of tuberculosis. More than 50 diagnostic tests are currently in development, but rigorous evaluation of the entire diagnostic process faces difficulties, such as the absence of quality control reagents. It is crucial to make progress in the development of appropriate phenotypic testing methods and robust quality assurance systems [98].

The collection and transport of *M. tuberculosis* specimens continue to be a challenge in settings where tuberculosis is widespread and the necessary infrastructure to maintain specimen integrity is lacking. A study addressed this issue, and PrimeStore Molecular Transport Medium (MTM) was developed, which not only rapidly inactivates *M. tuberculosis* but also preserves genomic DNA under high-temperature conditions, thus facilitating subsequent molecular analysis, which provides information essential for a correct diagnosis and adequate treatment of tuberculosis patients [99]. Molecular testing by Xpert MTB/RIF for tuberculosis could bring significant savings to health systems in high-income countries by decreasing the need for patient isolation and the total length of hospitalization [100].

One of the essential criteria for performance evaluation is the participation of laboratories in the external quality assessment (EQA) for the Xpert MTB/RIF test, which is integrated into the quality assurance system necessary for clinical practice and laboratory use. This system includes the pre-analytical, analytical, and post-analytical processes, with the aim of guaranteeing the continuous quality of the tests [101].

### 4.3. Loop-Mediated Isothermal Amplification (LAMP) Technology

Other molecular techniques include ligase chain amplification, for identifying drug resistance mutations, and LAMP, which is faster and less expensive than PCR [102,103].

LAMP is a fast and simplified NAAT platform developed by Eiken Chemical Co., Ltd. (Tokyo, Japan). The technology uses four different primers specifically designed to recognize six distinct regions of the target gene, and the reaction process takes place at a constant (isothermal) temperature using the strand displacement reaction. A simplified amplification test technique was used in one study loop-mediated isotherm (LAMP). The procedure was performed on a semisolid gel of polyacrylamide of dimensions 6 × 8, using a prototype device accessible from the financial point of view. Each serving of the gel contains a small amount of only 670 nanoliters, thus reducing the need for large amounts of chemicals. Amplified DNA is identified by means of the fluorescence of the LCGreen Plus+ dye embedded in the gel, along with other reagents [104].

Amplification and detection of gene products can be completed in one step by incubating the mixture of samples, primers, DNA polymerase with strand displacement activity, and substrates at a constant temperature. Amplification efficiency is high, and DNA can be amplified 10^9^–10^10^ times in 15–60 min. Due to its high specificity, the presence of the amplified product can indicate the presence of the target gene. Currently, there is limited evidence regarding the accuracy of LAMP for TB detection.

A study conducted in Gambia, compared various methods of detecting tuberculosis using sputum samples, both in patients with symptoms suggestive of TB and in patients confirmed with this disease. The loop-mediated amplification assay for TB (TB-LAMP) was evaluated in comparison with other techniques such as smear microscopy with ZN, MGIT culture, and GeneXpert MTB/RIF. The reference standard was culture. TB-LAMP showed an overall sensitivity of 99% and a specificity of 94%. In the latent class analysis, TB-LAMP had a sensitivity of 98.6% and a specificity of 99%, while GeneXpert had the highest sensitivity (99.1%) but the lowest specificity (96%). Both TB-LAMP and GeneXpert showed high sensitivity and specificity in detecting TB, regardless of age or strain of infection. These findings underline the utility of both methods, GeneXpert and TB-LAMP, in the diagnosis of TB. However, although TB-LAMP requires less infrastructure, it cannot detect drug-resistant strains, making it more suitable for the initial testing of new TB cases in medical clinics [105].

However, widespread implementation of these techniques in developing countries is still limited by a lack of infrastructure, high costs, and a lack of skilled personnel. In addition, the need for adequate transport and storage of samples is another important challenge.

Another study investigated the effectiveness of parallel tests for the diagnosis of pulmonary tuberculosis in patients whose smear results were negative. In this study, 258 patients were included, and different testing methods, including culture, GenXpert MTB/RIF, and SAT-TB, were compared. The results revealed that the use of parallel tests resulted in significantly higher sensitivity compared to individual testing. The parallel testing model demonstrated a significant improvement in diagnostic efficacy for smear-negative PTB. Thus, this method should be considered in clinical practice when PTB is suspected but smear results are negative [106]. The same LAMP is a rapid assay to prove the rifampicin and isoniazid resistance of TB isolates [107].

### 4.4. PCR Multiplex Seegene Anyplex MTB/NTM MDR-TB

The Seegene Anyplex MTB/NTM MDR-TB test is an innovative technology that offers a cutting-edge approach to the precise diagnosis of tuberculosis and nontuberculous mycobacterial infections (NTM). Developed by Seegene (Seoul, Republic of Korea), this test stands out for its ability to detect drug resistance (MDR-TB), thus providing crucial information to guide optimal patient treatment.

The Seegene Anyplex MTB/NTM MDR-TB molecular method uses a multiplex PCR system for the accurate detection of *M. tuberculosis* (MTBC), nontuberculous mycobacteria (NTM), and drug resistance. In a multicenter study, the Anyplex test showed a sensitivity of 86.4% for lung samples and 83.3% for extrapulmonary samples compared to the rapid acid bacillus smear (75.0% and 50.0%, respectively). Specificities were 99% and 99.4% for lung samples and 100% for extrapulmonary samples. In addition, Anyplex identified isoniazid resistance with a sensitivity of 83.3% and 100% specificity in both types of samples. The conclusion is that the Anyplex MTB/NTM MDR-TB test is effective for the diagnosis of pulmonary and extrapulmonary tuberculosis, including the detection of isoniazid resistance [108].

### 4.5. WGS Sequencing (Whole-Genome Sequencing)

WGS sequencing is a state-of-the-art technology that involves accurate determination of the order of nucleotide bases in the entire genome of an organism, including *M. tuberculosis* (MTB).

In 1998, the entire genome of the best-characterized strain of *M. tuberculosis*, H37Rv, was sequenced, marking an important milestone in research into this bacterium. This sequencing was followed by a detailed analysis, with the aim of understanding more deeply the biology of this slow-growing pathogen and contributing to the development of new prophylactic and therapeutic interventions. The MTB genome contains a total of 4,411,529 base pairs and approximately 4000 genes, showing an extremely high content of guanine and cytosine [109].

This method provides a comprehensive view of the bacterial genetic material, facilitating the identification of all genomic mutations and variations.

In the initial sequencing research, resistance to antituberculosis drugs was investigated, initially focusing on the specific *M. tuberculosis* genes associated with this resistance.

A study by Campbell et al. sequenced nine antibiotic resistance-associated locks in 314 clinical *M. tuberculosis* isolates, of which 52% were multidrug-resistant (MDR) and 3% were highly resistant (XDR).

Comparing the phenotypic data with the sequencing data, it was found that the sequence was able to accurately identify the resistance phenotype observed in the case of MDR isolates with a sensitivity and specificity of 90.8% and 94.7%, respectively. However, precise sequence-based determination of XDR isolates was more difficult, showing a sensitivity of only 40.0% and a specificity of 99.3% [110].

The WGS sequencing studies on drug-resistant isolates provided a better understanding of the mutations associated with treatment resistance but also highlighted the complexity of interpreting the data.

Regarding ethambutol resistance, research has shown that even low levels of resistance that would not normally be detected by traditional phenotypic antibiotic susceptibility tests can be accumulated in the *M. tuberculosis* genome [111].

In another study, it was revealed that phenotypic testing indicated isoniazid resistance, while sequencing the entire genome could not identify the associated mutation [112].

## 5. Diagnostic immunological tests

### 5.1. Urinary Lipoarabinomannan Test (TB-LAM)

The urinary lipoarabinomannan test (TB-LAM) is a diagnostic method used to identify infection with *M. tuberculosis*. This test focuses on the detection of a substance called lipoarabinomannan (LAM), which is part of the cell wall of the *M. tuberculosis* bacterium.

Lipoarabinomannan (LAM) is an immunogenic lipopolysaccharide found in mycobacterial cell walls, released from metabolically active or degenerative bacterial cells. It is found predominantly in people with active tuberculosis disease. The TB-LAM test exhibits low cross-reactivity, being largely specific to tuberculosis-like mycobacterial infections, with reduced interaction with nontuberculous mycobacteria [113].

The test procedure involves collecting a sample of urine from the patient, and then this sample is examined for the presence of LAM using specific techniques.

The LF-LAM (lateral flow lipoarabinomannan) test is performed manually by applying 60 μL of unprocessed urine onto the test sample buffer. After application, the test is allowed to incubate at room temperature for 25 min. This procedure allows for the rapid and effective identification of the presence of the specific LAM antigen *M. tuberculosis* in the urine, delivering results in a relatively short time interval [114].

If LAM is detected in urine in significant concentrations, this indicates a possible infection with *M. tuberculosis*.

In advanced stages of immunodeficiency, such as co-infection with HIV, the elimination and detection of lipoarabinomannan (LAM) in the urine is found [115].

This phenomenon leads to the systemic spread of *M. tuberculosis* and an increased mycobacterial load, caused by dysfunction of podocytes and changes in the rate of glomerular filtration. This situation contributes to a significantly higher concentration of the LAM antigen in the urine [116].

In accordance with the revised guidelines of the World Health Organization (WHO) on the use of the urinal lipoarabinomannan test (TB-LAM) for the diagnosis of tuberculosis (TB), it is reliably recommended to use this test in HIV-positive patients with a CD4 count of less than 100 cells/μL, regardless of the presence of signs and symptoms of tuberculosis [117].

A study conducted by Tobias Broger and colleagues evaluated the comparability of two urinal lipoarabinomannan-based fast-care-point diagnostic tests (POCs) for tuberculosis. The first commercial test, Alere Determine TB LAM Ag (AlereLAM), revealed that lipoarabinomannan concentrations are correlated with the severity of the disease and the risk of mortality in hospitalized HIV patients, improving the results.

However, this test shows moderate diagnostic sensitivity. By comparing it with the new Fujifilm SILVAMP TB LAM (FujiLAM) test, Broger and his colleagues found that FujiLAM has a higher diagnostic sensitivity (70.4% vs. 42.3%) without compromising specificity.

Based on these results, the authors concluded that the FujiLAM test improves diagnostic sensitivity without compromising specificity compared to the AlereLAM test.

The global health community now has two non-sputum biomarker tests that could be used at the care point to diagnose tuberculosis in people with HIV in endemic countries [118].

In a retrospective analysis study, a population of pediatric patients with severe acute malnutrition was investigated in a rural health center in Mozambique.

The samples collected, including sputum, pharyngeal secretions, and urine, were analyzed for mycobacterial culture and compared with the results of the Xpert MTB/RIF and TB-LAM tests. The TB-LAM test from urine samples revealed a good correlation with the clinical diagnosis of infant tuberculosis. These findings suggest that TB-LAM testing in the urine may be an effective and relevant way for early diagnosis of tuberculosis among children with severe acute malnutrition [117].

Another study conducted in Ethiopia, the country with one of the highest rates of extrapulmonary tuberculosis (EPTB), conducted an evaluation of the diagnostic performance of the Xpert MTB/RIF test and the TB-LAM test for a rapid diagnosis of extrapulmonary TB (EPTB). The TB-LAM test itself shows reduced sensitivity in the diagnosis of extrapulmonary tuberculosis (EPTB). However, the combination of TB-LAM and Xpert MTB/RIF significantly improves EPTB diagnosis, especially in countries with a high prevalence of EPTB and co-infection with HIV [119].

### 5.2. Gold Nanoparticles (AuNPs)

Gold nanoparticles are among the most promising tools in the field of biosensing due to the numerous physicochemical properties derived from the nanoscale, which include enhanced spectroscopic signals and enzyme-mimicking properties [120].

Advances in nanotechnology open new perspectives for the development of rapid, sensitive, and cost-effective sensors for the detection of *M. tuberculosis*. The TB disease caused by *M. bovis* is clinically and pathologically indistinguishable from TB caused by MTB. In addition, both mycobacteria stain as acid-fast bacilli, are 99.95% genome-wide similar and have identical 16S rRNA sequences. Differentiating between them is crucial because *M. bovis* is intrinsically resistant to pyrazinamide, one of the commonly used first-line anti-TB drugs [121].

In one study, an innovative immunoassay was created and evaluated using ferromagnetic gold nanoparticles to detect and differentiate the main causative agents of human tuberculosis, namely MTB and *M. bovis*. By generating a single recombinant monoclonal antibody directed against a key MTBC-specific protein and combining it with a range of pre-existing antibodies targeting both cell surface and secreted antigens of MTB and *M. bovis*, the proposed assay was developed. NP bioconjugates, consisting of Au-Fe_3_O_4_ ferromagnetic gold nanoparticles, were obtained by direct binding of antibodies to these particles [121].

### 5.3. Interferon-Gamma Release Tests (IGRA)

Interferon-gamma release tests (IGRA) are used in the diagnosis of *M. tuberculosis* infection (MTB), including latent tuberculosis (LTBI). These tests measure the body’s immune response to specific MTB antigens, such as the proteins ESAT-6 and CFP-10, which are absent in the BCG vaccine and in most nontuberculous mycobacterial strains.

For the detection of latent infection with *M. tuberculosis* (LTBI), the skin test for tuberculin (TST) is the preferred method. However, it cannot be considered the gold standard due to the significant number of false positive and false negative results, as well as the variability of their interpretation [122].

A new generation of tests has been developed, such as the QuantiFERON-TB Gold (QFN-GOLD) and the ELISpot test (also known as the T-Spot TBC).

These tests are based on serum detection of interferon-gamma (INF-γ) released by stimulation of T cells sensitized by the *M. tuberculosis* in vitro antigen (QuantiFERON) using a full-blood enzyme-linked immunosorbent test (ELISA) or direct detection for T cells (ELISpot) [123].

These tests are currently an advantageous alternative to TST in terms of the limitations mentioned above and have proven to be promising as alternative diagnostic tools for latent tuberculosis (LTBI) in BCG-vaccinated populations [124].

However, IGRA tests face several challenges in identifying active, latent, and cured tuberculosis infections, as well as in diagnosing this infection due to the need to incubate clinical samples overnight and the complexity of measuring the level of interferon (IFN)-γ [125].

One study examined the QuantiFERON-TB Gold In-Tube Test (QFT-G IT) and investigated the clinical and laboratory factors that influence the rate of undetermined results of this test. Immunosuppressive therapy, underlying diseases, bed immobilization, and hypoalbuminemia were significantly associated with uncertain QFT-G test results. However, a delay of more than 6 h in the incubation process increased the frequency of undetermined results. This research highlights the importance of optimizing test procedures to reduce rates of undetermined results, which could contribute to an earlier diagnosis of tuberculosis [124].

Another multicenter retrospective cohort study was conducted on a sample of 1264 patients diagnosed with culturally confirmed tuberculosis. These patients were subjected to the QuantiFERON-TB Gold In-Tube test, designed to detect *M. tuberculosis* infections, whether latent or clinically manifested.

Information on host factors that could contribute to false negatives or undetermined results is limited. However, several host factors have been identified, such as old age, the presence of widespread pulmonary tuberculosis, a malignancy, and lymphocytopenia, which can be associated with negative results in the QuantiFERON-TB Gold In-Tube test in patients with culturally confirmed tuberculosis [126].

QuantiFERON-TB Gold Plus (QFT-Plus) is a modern test used to diagnose tuberculosis (TB). This is an improvement to the original QuantiFERON-TB Gold In-Tube test (QFT-GIT). QFT-Plus uses three MTB-specific antigens: ESAT-6, CFP-10, and TB7.7 (Rv2654c). They are combined with the components of the patient’s blood in the test tubes.

A study conducted at a medical center in Tokyo, Japan, examined 99 patients with laboratory-confirmed active tuberculosis and 117 healthy volunteers, without risk of tuberculosis infection, who served as a control group. Blood samples were collected from both groups and tested using three types of IGRA (interferon-gamma release assays): QFT-Plus, QuantiFERON-TB Gold In-Tube (QFT-GIT), and T-SPOT TB (PUNCTUL T). The sensitivity and specificity of each IGRA were evaluated and compared in this study.

The results showed that QFT-Plus demonstrated a significant degree of consistency with QFT-GIT and T-SPOT, showing both high sensitivity and specificity. These findings suggest that QFT-Plus can be an effective tool for diagnosing tuberculosis, providing a reliable and accurate alternative for the detection of this infectious disease [127].

## 6. Conclusions

The previously described methods for the diagnosis of tuberculosis, such as molecular tests and the microscopic method, as well as bacterial culture, play an essential role in the effective detection and management of this serious disease. While many of these methods of diagnosis and treatment are promising, it is essential to continue research and develop more effective and cost-effective approaches to the management of tuberculosis. In particular, more studies are needed that focus on developing and testing new drugs and therapies, improving screening and diagnostic strategies, and improving approaches to TB control and prevention.

Nevertheless, the microscopic method remains a widely used diagnostic technique, especially in countries with limited resources. However, it is important to recognize the limitations of this method and consider the use and evaluation of other techniques to obtain a complete and accurate diagnosis.

Bacterial culture, both in solid and liquid media, is an essential method in the diagnosis of tuberculosis. Culture on solid media provides specificity and accuracy, while culture on liquid media brings speed and increased sensitivity. The use of these methods in combination can contribute to a complete and accurate diagnosis of tuberculosis.

Molecular diagnostic techniques have represented a major leap forward in the detection and management of tuberculosis. They enable the rapid identification of *M. tuberculosis* complex and drug resistance, thus providing critical information for choosing the appropriate treatment regimen. Molecular tests, such as LPA and GeneXpert MTB/RIF, offer significant benefits in the rapid and accurate diagnosis of tuberculosis, including drug-resistant forms. These tests allow the identification of resistance mutations and provide essential information for choosing the right treatment. Thus, molecular techniques are an essential tool for rapid and accurate diagnosis of tuberculosis and drug resistance. Although molecular tests are extremely useful, they also have important limitations. For example, these tests cannot distinguish between viable and nonviable *M. tuberculosis* complexes, which means they are not suitable for monitoring treatment response. However, their widespread implementation in countries with limited resources requires a comprehensive approach that takes into account all specific challenges and needs.

In general, combined diagnostic methods, which use several techniques and approaches, give the best result in the diagnosis of tuberculosis. Improving the quality and accessibility of tests, as well as the implementation of advanced technologies, can help improve the sensitivity, efficiency, and accuracy of tuberculosis diagnosis.

It is essential that we continue research and development of new methods and technologies to improve the diagnosis and management of tuberculosis given its significant impact on global health.

## Data Availability

No new data were created or analyzed in this study. Data sharing is not applicable to this article.
